# Mechanochemistry in Europe: where we come from and where we are now

**DOI:** 10.12688/openreseurope.19848.1

**Published:** 2025-03-20

**Authors:** Juan José Sáenz de la Torre, Leyre Flamarique, Fernando Gomollon-Bel, Evelina COLACINO

**Affiliations:** 1 Agata Communications, Santander,, Spain; 2 ICGM, Univ Montpellier, CNRS, ENSCM, Montpellier, France

**Keywords:** Mechanochemistry, Green Chemistry, Sustainability, European Green Deal, solvent-free methods, eco-friendly processes, policy, standardisation

## Abstract

Mechanochemistry is several thousand years old, but it has been overlooked in favour of solution-based chemistry for long time and its methods have only recently emerged as a key technology for advancing green and sustainable chemistry, offering a solvent-free (or low-solvent) alternative to traditional chemical processes. Successfully used in metallurgical industry, mechanical alloying paved the way to the use of mechanochemistry in other industrial sectors ( *e.g.*, for waste treatment and soil decontamination), with potential applications also for preparing pharmaceuticals, transforming biomass, recycling and degrading polymers, just to cite some. Overall, it seems to provide an interesting pathway to decarbonizing the chemical industry. Several initiatives have subsequently blossomed under the combined efforts of scientists belonging to the International Mechanochemical Association (IMA): the approval of the COST Action CA18112 MechSustInd and the consequent building of collaborative networks favoured the funding of the EU Horizon IMPACTIVE project, the establishment of the EuChemS Working Party on Mechanochemistry, the organization of the Round Robin project on mechanochemical transformations and the approval of the IUPAC project on terminology and symbolisms. All these initiatives are actively promoting mechanochemistry by addressing challenges in the field and fostering collaboration across academia and with industry. These initiatives place Europe at the forefront of mechanochemical innovation, pushing forward environmentally friendly chemical processes while fostering the development of standardised terminology and industrial applications.

Mechanochemistry is the science of building and breaking down bonds by the “ *direct absorption of mechanical energy*” ^1^, where transformations are driven by mechanical forces applied by impact, shear, compression, or tension. The use of mechanical forces for promoting a chemical transformation were reported in a fragment of the ancient work ‘ *De lapidibus (On stones)* of Theophrastus of Eresus (4 ^th^ century BC), which is the oldest fragment found so far concerning chemical sciences. Indeed, the obtention of metallic mercury is described by rubbing and grinding of its mineral, cinnabar, in a copper mortar using a pestle of the same material ^2, 3^.

Even though, over the times, solution-based chemistry has become the industrial standard, in the last decades the number of publications on mechanochemistry has multiplied greatly, with applications in several fields ( Figure 1), but with a different stage of maturity of the technology, depending on the industry sector mechanochemical processing is applied to ^4^. Defined by the Technology Readiness Levels (TRL) ^5^, the highest TRL (TRL 9) is for applications in the field of energy and hard materials ( *e.g.*, metallurgy and waste management, just to cite some), for which mechanochemistry is a competitive manufacturing process.

**Figure 1.**
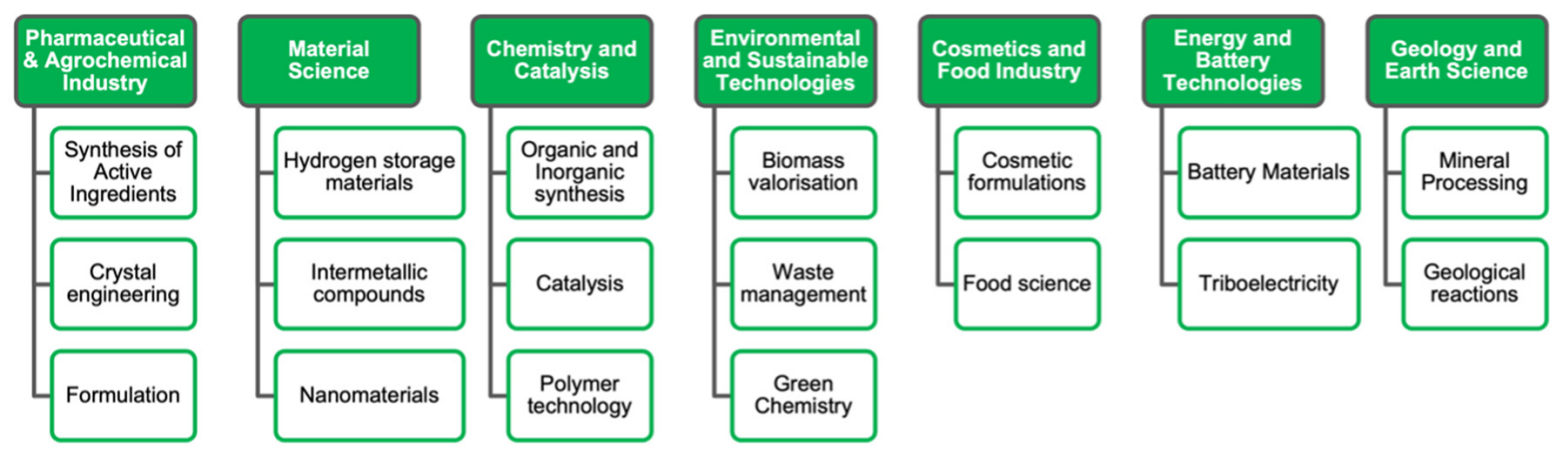
Application of mechanochemical processing.

In a context where the demand for sustainable reaction conditions and cleaner processes is increasing globally, mechanochemistry was identified in 2019 by IUPAC among the ‘ *emerging technologies to make our planet more sustainable’* ^6^ and, in 2021 as a powerful tool for achieving the United Nations' Sustainable Development Goals (UN SDGs) ^7^, and the European Green Deal objectives ^8^. Aligned also with the 12 Principles of Green Chemistry and Green Engineering, categorized based on their relevance to the upscaling of mechanochemical processes ^9^, mechanochemistry represents a groundbreaking approach ^10– 12^ able to foster green transition and sustainability, with the potential to decarbonise and reduce the environmental impacts of the chemical industry ^10, 12^.

Throughout Europe, there are many initiatives that, starting from the EU Programme COST Action CA18112 ‘Mechanochemistry for Sustainable Industry’ (MechSustInd), have contributed to highlight the potential of mechanochemistry, each one tackling different aspects in the field: some of them, like the Horizon Europe Project IMPACTIVE, are pushing forward the implementation of this technology in specific industrial settings, such as pharmaceuticals ^13^, the IUPAC Task group on ‘Terminology and Symbols for Mechanochemistry’ and the EuChemS Professional Network (PN) on mechanochemistry are transversal to both private and public sectors, while the Round Robin project pursues specific issues of fundamental research. This creates the conditions for cross-fertilisation of research areas, also aimed to create generally recognised scientific standards and guidelines for the field. Altogether, this set of initiatives, all of them involving members of IMA and, thus, academia, put Europe at a unique position to innovate in mechanochemistry, pushing forward a greener and more sustainable way of performing chemistry, contributing to the mentality shift for ‘ *thinking chemistry differently’* ^14^. In the next paragraphs, together with a foundational importance given to IMA, the different European initiatives dealing with mechanochemistry will be described in chronological order.

## International Mechanochemical Association (IMA)

The International Mechanochemical Association (IMA) is the first international cluster created around mechanochemistry. The association was founded in 1988 at Tatranská Lomnica (current Slovakia), with representatives from Japan, and former USSR and Czechoslovakia. Currently, the association counts 120 members distributed across 31 Countries in 4 continents. Over the years, IMA built both geographic and scientific bridges, merging the expertise of scientists, pioneers in the field, advancing the deep understanding of mechanochemical processing applied to hard materials and alloying. This has crystallised in numerous collaborations and initiatives and has paved the way towards the growth of the field, leading to further implementation of mechanochemical methods also in other areas of chemistry and process engineering.

Another strong pillar of IMA is its focus on young scientists, to teach and train them on mechanochemical methodologies and its flagship initiative is INCOME, the International Conference on Mechanochemistry and Mechanical Alloying occurring every three years. The first edition of INCOME took place in Kosice (Slovakia) in 1993. Since then, there has been 10 editions of the conference, that grew in numbers and impact over the years. The 11 ^th^ INCOME Conference will take place in Berlin-Adlershof (Germany) in 2025, bringing together hundreds of researchers in mechanochemistry from all over the world.

More information about IMA is available at imamechanochemical.com

## MechSustInd: a COST action focused on mechanochemistry

The several facets of mechanochemistry converged more recently in the European Programme COST Action CA18112 ‘Mechanochemistry for Sustainable Industry’ (MechSustInd) active between 2019 and 2023 ^15– 18^. Even after its closure, the foundational successes, innovative approaches, and concepts that emerged from the COST Action CA18112 continue to expand and thrive beyond its duration, also inspiring similar initiatives worldwide.

Promoting the European Cooperation through Science and Technology, the Action was dedicated to establishing scalable processes, harmonizing nomenclature and guidelines, standardising terminology and protocols (to address industrial demands), facilitating knowledge sharing, equipping researchers and emerging scientists with the necessary skills to incorporate sustainable practices through mechanical activation into daily research activities, and advancing the cooperation between industrial partners and academic researchers, raising awareness on the advantages of the integration of mechanochemistry into chemical processes at both R&D phase and production scale.

Through the several networking and scientific initiatives organized all along its lifetime, the Action has harmonized fundamental and applied research with technological innovation and industrial needs in mechanochemistry, attaining the far-reach objectives connected with the development of green economy for the sustainable production also of fine chemicals and pharmaceutically relevant molecules ^19^.

 MechSustsInd was able to establish the formation of a multi-disciplinary network (going beyond the European borders) of scientists, engineers, entrepreneurs, and investors with expertise encompassing different areas of mechanochemistry, and distributed across three different Working Groups ^20^, addressing both lab-scale (WG1) and up-scaling (WG3) mechanochemical syntheses via technology assessment, providing mechanistic investigations behind the reactions in the other WGs (WG2), and quantitatively assessing them for their advantages over existing solution-based methods. The use of green chemistry metrics, and through unprecedented studies and methods for life cycle assessment (LCA) and techno-economic analyses (TEA) applied to a ‘case study’ for the mechanochemical synthesis of a World Health Organisation (WHO) essential medicine ^21^, the COST Action had a specific ambition to get mechanochemistry closer to industry.

**Figure 2.**
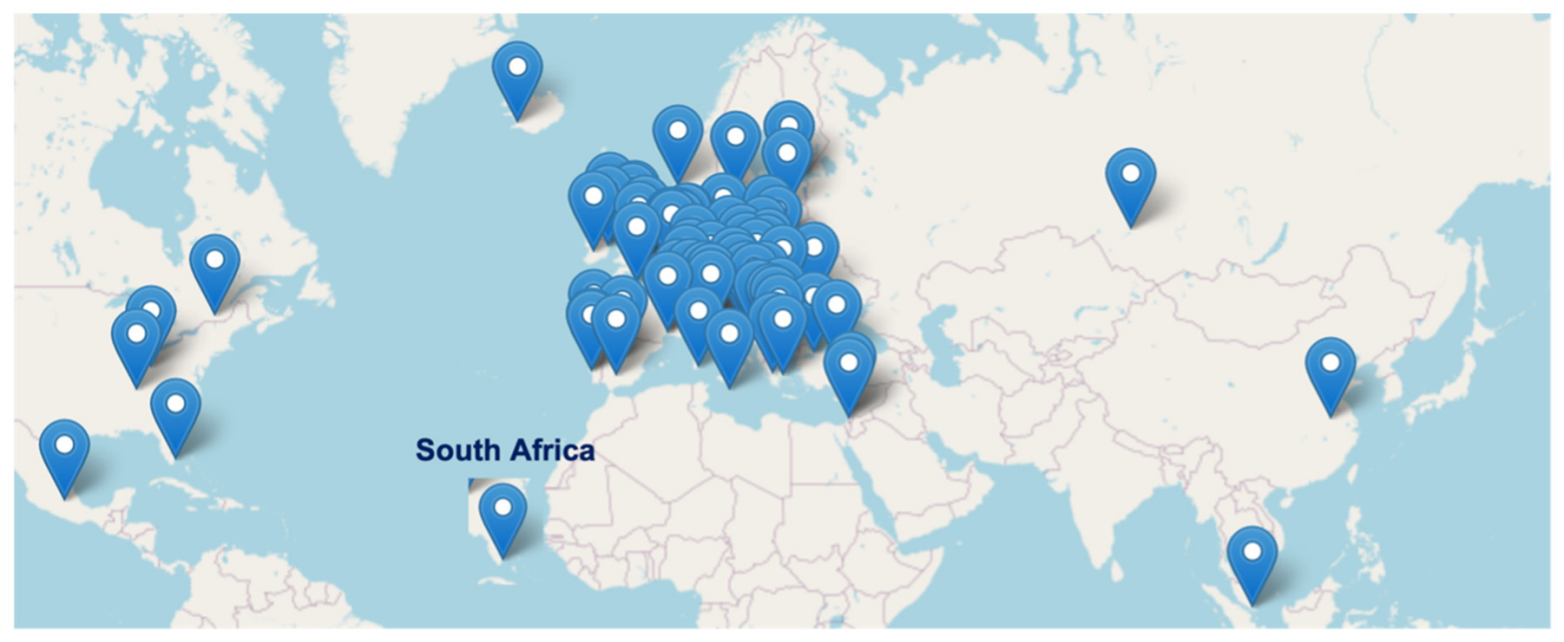
Geographic distribution of CA18112 members, beyond European borders.

 The initiative counted 150 members, scientists active in both public and private sectors and operating in 46 participating countries (38 in Europe), also beyond the geographical boundary of Europe, including among its members, scientists based in Canada, China, Japan, Mexico, Russia, Singapore, South Africa, South Korea, and USA, ( Figure 2).

Despite the disruption caused by the worldwide pandemic, MechSustInd reached high level of achievement also in fostering the integration of mechanochemistry in educational activities and academic curricula, answering the EU needs of 'new skills' for 'new professions' in green chemistry field, for the young generations of scientists.

This unprecedented output for the field, materialised by the organisation of five international training schools across Europe, with speakers from both academia and industry, by the integration of complementary expertise from diverse stakeholders and covering key topics such as: i) mechanochemical organic synthesis and kinetics (in Italy), ii) up-scaling mechanochemical reactions (in Germany), iii) *ex-situ* and *in situ* analyses of mechanochemical reactions (in Croatia and webcasted from Malta), and iv) structural characterization of supramolecular and covalent compounds (webcasted from Portugal).

The pioneering implementation of mechanochemistry in teaching had a remarkable outreach at international levels, and beyond the European geographical borders. Indeed, MechSustInd bridged mechanochemistry with green chemistry education: i) by creating a strategic partnership with internationally recognised associations ( *e.g.*, Beyond Benign, USA) ^22^ that foster education in green chemistry and sustainability, ii) published the first educational article on mechanochemistry ^23^, and iii) released two educational books on the same topic ^24, 25^.

 Pandemic was turned in an opportunity: since 2020 and for the entire period of lock-down, 31 webinars involving 53 speakers from Europe and worldwide, animated the biweekly webinar series ‘ *Get together during COVID’*. This unique approach allowed cross-fertilisation of expertise, kept the strong connections between the members and advanced the community building objectives.

Strategic liaisons/partnerships could be established with the decision bodies of National and European Chemical Societies and Chemical Associations across Europe. Notably, strong connections were built with: i) other COST Actions ( *e.g.*, CA18224 Greenering and CA19122 EUGAIN), ii) the International Mechanochemical Association (IMA) to foster the interaction among scientists having pioneered the field of mechanochemistry and the 'new comers' implementing mechanochemistry in areas not yet explored, and iii) key stakeholders and agencies for standardisation, and iv) the EuChemS Professional Network for Young Chemists' ^26^.

Remarkably, jointly with EYCN, MechSustInd implemented the first edition of an international award for an emerging young scientist active in the field of mechanochemistry, recognising excellence through competitions.

The groundbreaking concepts, the impactful scientific directions (also witnessed by more than 100 research outputs, press releases, editorials, special issues, etc.) and the successful activities that emerged from the COST Action CA18112 are still under development, continuing to flourish after its lifetime and beyond the European geographical borders. It would be unrealist to sum them up herein in few lines. Only few of them were herein described, as a contribution to the further development of mechanochemistry field.

In a recent study on impact assessment of COST Actions ^27^, CA18112 was highlighted as a ‘case study’ for innovation, based on criteria evaluating its structure and execution, as well as the range of creative outputs it led, including business models, new products (such as patents), and educational formats, while enhancing communication with policymakers, key stakeholders and the public. This is particularly rewarding considering the disruption caused by Covid-19 pandemic, heavily impacting the implementation of activities for which networking and experimental activities were inescapable needs.

More information can be found at mechsustind.eu/

## IMPACTIVE: a Horizon Europe project dedicated to mechanochemistry in pharmaceutical industry

**Figure 3.**
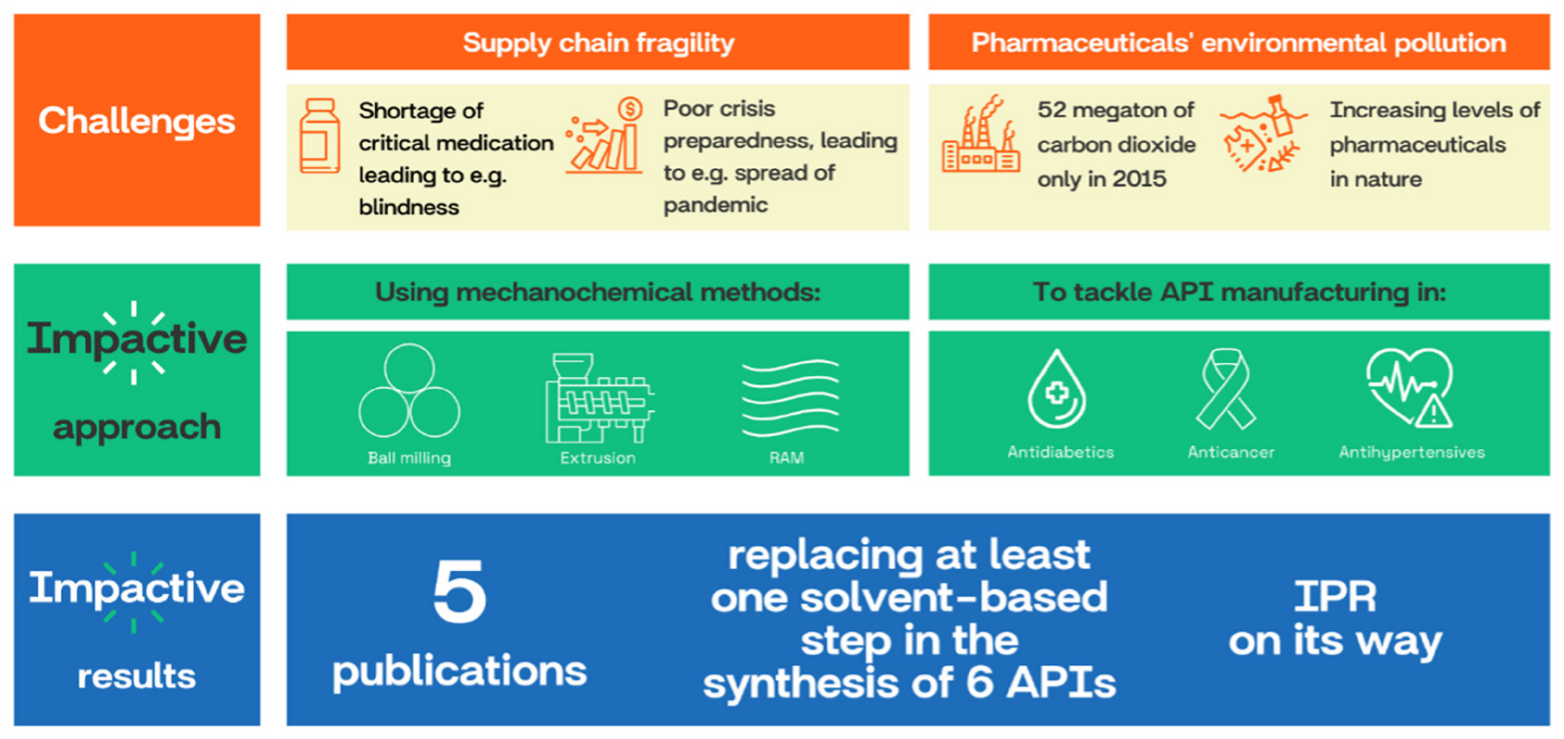
IMPACTIVE project’s summary and objectives.

IMPACTIVE (Innovative Mechanochemical Processes to synthesize green ACTIVE pharmaceutical Ingredients) project, funded under Horizon Europe, focuses on applying mechanochemical methods and processes to API manufacturing ^13^. It aims to provide proof-of-concept at a small pilot scale of the use of batch and/or continuous flow mechanochemistry to produce 6 APIs from 4 different families of compounds ( Figure 3). The 4-years project has started in October 2022, and it brings together 17 partners operating across 11 European countries. It includes universities and research institutions, SMEs and two major players in pharmaceutical industry ( *e.g.*, Novartis and Merck). The consortium members are skilled with diversified backgrounds covering research, development, manufacturing and exploitation.

The obstacles of applying mechanochemistry to produce Active Pharmaceutical Ingredients are not always of technical nature. Among R&D managers of the chemical and engineering fields, mechanochemistry is still highly unknown ^28^, which slows down its adoption for commercial purposes. The barriers for and the advantages of industrial mechanochemistry, also in terms of sustainable finance, were highlighted previously ^9^. However, mechanochemistry provides three main advantages that are worth exploring:

1.   In a chemical process, solvents make up to 90% of the reactant mass ^29^, and most of the solvent used in chemical reactions and manufacturing processes are far from being environmentally friendly ^30, 31^. Therefore, removing the solvents completely or using them in a minimum amount (as for Liquid-Assisted Grinding processes, LAG) ^32^ — leads to greener syntheses ^33^ with better green chemistry and environmental metrics ^34^. For API manufacturing, the advantages are game-changing. For a ‘case study’, indicators such as ecotoxicity and CO _2_ emissions could be reduced to 85% ^19, 35^.

2.   When working at an industrial scale, heating and cooling the masses is not a trivial issue, and involve additional costs ^36^. Since mechanochemistry methods operate at lower temperatures, the operating costs for API manufacturing can be possibly reduced. Again, for a ‘case study’ the production costs were decreased by 12 % ^21^.

3.   As reported both in media ^37^ and by the European Commission ^38^, supply chains have become subject to disruptions and shortages. The strict regulations adopted to achieve the EU Green Deal objectives prompted API manufacturers to relocate production elsewhere, resulting in EU pharmaceutical industry dependence on foreign supply chains. By creating more sustainable and safer processes for the chemical and pharmaceutical industries, mechanochemistry can help in bringing back the production to EU ( Figure 3), complying with the highest environmental standards enforced in Europe and not without the recognition of this sustainable technology ^6^ by health authorities, regulators and policy makers.

More information about IMPACTIVE can be found at https://mechanochemistry.eu/

## Round Robin project on mechanochemistry

 Officially started in 2024, the Round Robin project involves 32 participants from 15 research institutions. Most of the laboratories involved is European and several principal investigators have also participated in the COST Action CA18112. It represents the joint research efforts of researchers aimed to study the reproducibility of mechanochemical reactions and their standardisation by identifying the mechanochemical variables and conditions that allow to control a reaction. The objective is to show that mechanochemistry is reproducible as far as steady states are considered, regardless the equipment used and the experimentalist at work. The project focuses on the investigation of 3 chemical reactions, 1 co-crystal formation and 1 polymorphic transformation, distributed across the different research groups to test the validity of protocols and verify a few basic working hypotheses.

## IUPAC task group on terminology and symbolism for mechanochemistry

In 2019, the International Union of Pure and Applied Chemistry (IUPAC) identified continuous flow mechanochemical processes among the ten emerging technologies to make the planet more sustainable ^6^.

Although mechanochemistry has deep historical roots ^2, 3^, it lacks detailed and standardised terminology: tribochemistry, mechanical alloying, mechanochemistry… All these names refer to the application of mechanochemical force to induce chemical reactions. The term chosen for this article (mechanochemistry) is itself weakly defined in the *IUPAC Compendium of Chemical Terminology* ^1^. It describes a mechanochemical reaction as one ‘ *induced by the direct absorption of mechanical energy.*’

When joined with the wide range of materials, equipment, methods, and practices involved in mechanochemical processes, harmonised definitions are a need, filling the gap in terms of standardization and clarity in mechanochemistry’s terminology and symbolism. An initiative directly stemming from the COST Action CA18112 activities contributing to fill this gap and to move a step forward towards the growing recognition of the field occurred in 2024, with the IUPAC supporting the creation of a Task Group on ‘Terminology and Symbolism for Mechanochemistry’ ^39^. The project aims to establish uniformity in the terminology and classifications employed in mechanochemistry, proposing standardised symbolism and terminology to facilitate communication within the mechanochemical community and fostering the adoption of mechanochemistry, not only in academia, but also in R&D activities and industrial manufacturing. The mid-term goal is to provide objective guidelines finding the consensus of the international community, to advance the field, while enhancing its visibility and credibility towards different stakeholders.

More information about the IUPAC Project on Terminology and Symbolism for Mechanochemistry can be found at iupac.org/project/2023-034-2-100.

## EuChemS working party on mechanochemistry

12 ^th^ October 2023 represents a landmark for mechanochemistry in Europe and a unique initiative at global level: the creation of the “Working party on mechanochemistry” by the European Chemical Society (EuChemS) took place in Larnaca (Cyprus). This professional network (PN) represents a further implementation of the COST Action CA18112 'Mechanochemistry for Sustainable Industry', and it was created upon the strong willingness of its former members. Officially started in 2024, it counts already 32 members delegated by 22 National Chemical Societies across Europe, geographically well-balanced. Its members possess diverse expertise across various fields of mechanochemistry applications. The number of members continues to grow, reflecting the increasing interest in this innovative approach to " *thinking chemistry differently*" ^14^.

The main objective of the newly created PN on mechanochemistry within EuChemS is to serve as an ecosystem for science and innovation to enhance the visibility and integration of the field within the European scientific community and beyond.

**Figure 4.**
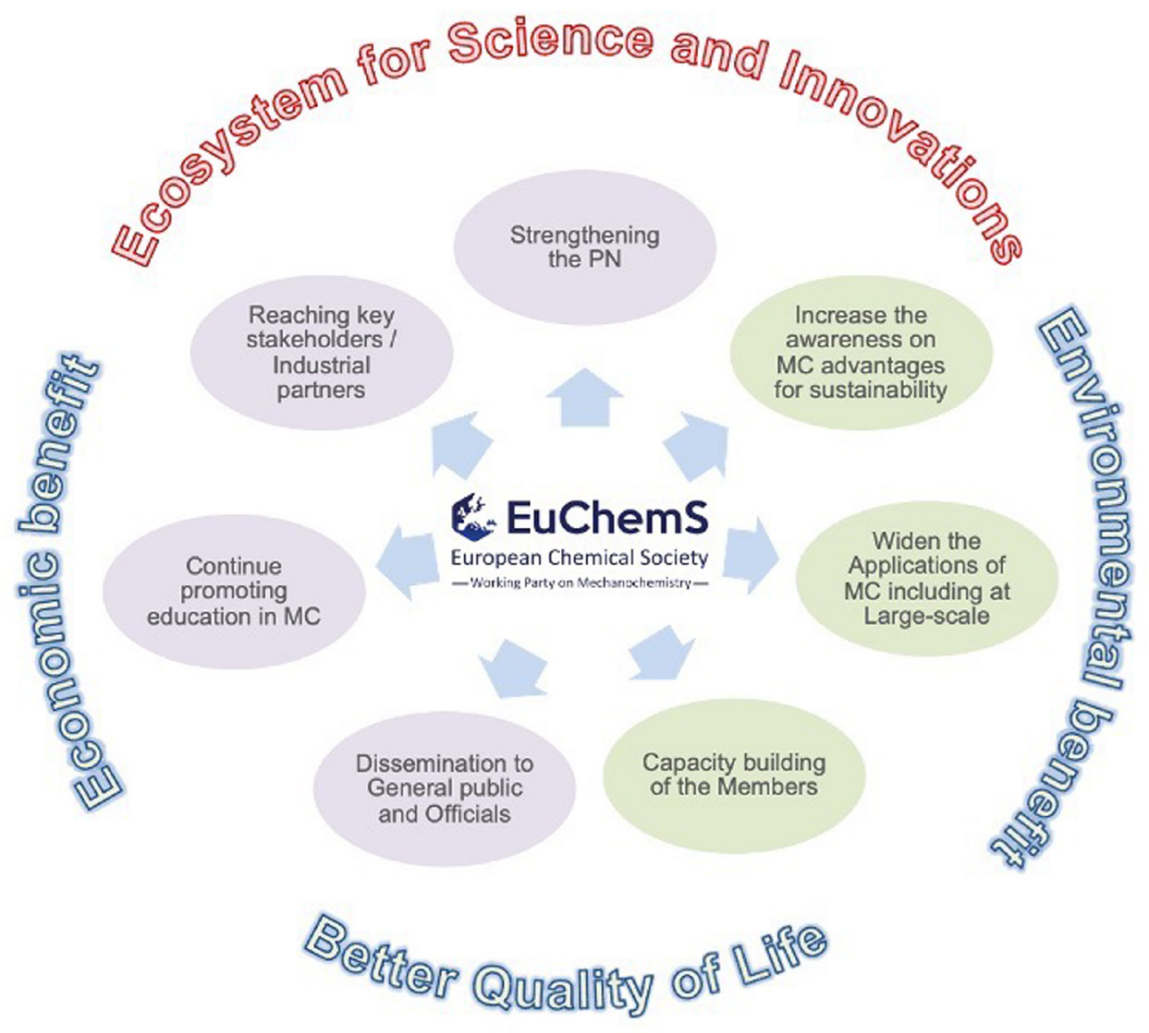
EuChemS Working Party on Mechanochemistry, aims and tasks. *Legend*: MC: mechanochemistry. PN: Professional Network.

Among the goals ( Figure 4), the Working Party on Mechanochemistry aims to: i) establish itself as the primary platform for discussion and knowledge exchange, advancing the understanding and significance of mechanochemistry and its principles. This will enable the reduction of the adverse impacts of traditional chemical and energy production by transitioning to more sustainable technologies, contributing to the achievement of the United Nations' 17 Sustainable Development Goals; ii) advocate for the broad adoption of mechanochemistry across fundamental and applied chemistry, as well as related interdisciplinary fields, by integrating it into academic and research institutions, industry, professional practices, and educational programs; iii) encourage and assist National Chemistry Societies in advocating for the significance, role, and advancement of mechanochemistry within their respective countries; iv) strengthen the role and credibility of mechanochemistry within the chemical sciences by engaging the general public and policymakers through social media, newsletters, and the organization of conferences and workshops accessible to society and key stakeholders; v) foster collaboration and networking among national groups and divisions of EuChemS member societies involved in chemistry and beyond, as well as with relevant European and international organizations; and vi) recognize excellence through competitions and awards.

This is a non-exhaustive list and more information will be regularly updated via its official webpage ^40^.

## Conclusions

In conclusion, these 6 milestones are just a selection of the myriads of initiatives related to mechanochemistry all over the world, creating a vibrant culture within the topic: from conferences, lectures, webinar series, international grants and targeted industrial applications. Mechanochemistry is a blooming field and has the potential to become a key tool in decarbonising chemical routes by providing processes that avoid or reduce the use of harmful solvents and beyond. This goal will be possible thanks to the collective work of the thousands of scientists and stakeholders involved in pushing mechanochemistry forward.

## Disclaimer

The views expressed in this article are those of the author(s). Publication in Open Research Europe does not imply endorsement of the European Commission.

The authors are part of the EU Horizon project IMPACTIVE, one of the milestones highlighted in this article.

## Data Availability

No data are associated with this article.
