# Supplementary material for: Mechanochemistry in Europe: where we come from and where we are now
Source: Open Res Eur. 2025 Mar 20;5:73. [Version 1] doi: 10.12688/openreseurope.19848.1 (PMC12485325; doi:10.12688/openreseurope.19848.1)
Supplement: Peer review history

# Reviewer report: Reviewer response for version 1

**DOI:** 10.21956/openreseurope.21472.r60844

**Published:** 2025-10-13

**Authors:** Juliana Vidal

**Affiliations:** 1 Beyond Benign Inc, Wilmington, Massachusetts, USA

The ' *Mechanochemistry in Europe: where we come from and where we are now' *is a well-written, accessible open letter that provides a brief history of the mechanochemistry field and a thorough summary of mechanochemistry initiatives and their impact on the European scientific community. The impacts and initiatives highlighted go beyond the academic sector, include training future scientists, and further emphasize the importance of collaborations between industry and academia to promote an impactful change.

Where applicable, are recommendations and next steps explained clearly for others to follow? (Please consider whether others in the research community would be able to implement guidelines or recommendations and/or constructively engage in the debate)

Yes

Does the article adequately reference differing views and opinions?

Yes

Are all factual statements correct, and are statements and arguments made adequately supported by citations?

Yes

Is the rationale for the Open Letter provided in sufficient detail? (Please consider whether existing challenges in the field are outlined clearly and whether the purpose of the letter is explained)

Yes

Is the Open Letter written in accessible language? (Please consider whether all subject-specific terms, concepts and abbreviations are explained)

Yes

Reviewer Expertise:

My area of expertise is in green chemistry, including mechanochemistry, biomass valorization, and polymer synthesis.

I confirm that I have read this submission and believe that I have an appropriate level of expertise to confirm that it is of an acceptable scientific standard.

# Reviewer report: Reviewer response for version 1

**DOI:** 10.21956/openreseurope.21472.r60839

**Published:** 2025-09-30

**Authors:** Marta Pineiro

**Affiliations:** 1 Department of Chemistry, CQC-IMS, University of Coimbra,, Coimbra,, Portugal

The Open Letter, “Mechanochemistry in Europe: where we come from and where we are now”, offers a valuable and clear overview of significant European mechanochemistry initiatives, effectively linking them to sustainability objectives such as the European Green Deal and the UN SDGs. The authors successfully trace the historical roots of mechanochemistry and demonstrate its modern industrial relevance, presenting Europe as a leader in the field.

 The review is written in a concise and accessible style, appealing to both specialists and non-specialists alike. It offers comprehensive coverage of six milestone initiatives: the International Mechanochemical Association (IMA), COST action MechSustInd, the Horizon Europe Project IMPACTIVE dedicated to mechanochemistry in the pharmaceutical industry, the Round Robin project, the IUPAC task group, and EuChemS working party on mechanochemistry. The text achieves an equilibrium between research and industrial applications, citing valuable pharmaceutical case studies. It places a strong emphasis on education, outreach, and community building.

 The review is Eurocentric in nature and therefore, with a lack of representation of initiatives from outside Europe. The use of quantitative data is predominantly case-specific. Furthermore, the incorporation of a forward-looking section that presents a critical analysis of realistic future directions, sector readiness, and the policy incentives required to support progress would be a valuable addition to the text.

Where applicable, are recommendations and next steps explained clearly for others to follow? (Please consider whether others in the research community would be able to implement guidelines or recommendations and/or constructively engage in the debate)

Yes

Does the article adequately reference differing views and opinions?

Yes

Are all factual statements correct, and are statements and arguments made adequately supported by citations?

Yes

Is the rationale for the Open Letter provided in sufficient detail? (Please consider whether existing challenges in the field are outlined clearly and whether the purpose of the letter is explained)

Yes

Is the Open Letter written in accessible language? (Please consider whether all subject-specific terms, concepts and abbreviations are explained)

Yes

Reviewer Expertise:

Organic Chemistry, Green Chemistry, synthesis of heterocycles, photophysical properties of heterocycles

I confirm that I have read this submission and believe that I have an appropriate level of expertise to confirm that it is of an acceptable scientific standard.

# Reviewer report: Reviewer response for version 1

**DOI:** 10.21956/openreseurope.21472.r52331

**Published:** 2025-04-03

**Authors:** José Berrocal

**Affiliations:** 1 University of Bristol, Cantock's Close,, Bristol, UK

The Open Letter by Colacino, Gomollon-Bel, and colleagues provides a valuable historical perspective on European activities in the field of mechanochemistry and emphasizes the potential of this field to decarbonize the chemical industry. The manuscript outlines the goals and contributions of six mechanochemistry initiatives that the authors consider milestones in the field. These include the International Mechanochemical Association (IMA), the COST Action "Mechanochemistry for Sustainable Industry" (MechSustInd, CA18112), the IMPACTIVE project ("Innovative Mechanochemical Processes to synthesize green ACTIVE pharmaceutical Ingredients") under Horizon Europe, the Round Robin project on mechanochemistry, the IUPAC task group on terminology and symbolism for mechanochemistry, and the European Chemical Society (EuChemS) working party on mechanochemistry.

 The manuscript highlights the pivotal role played by European institutions and researchers in these initiatives, as well as their collaboration with scientists from the former Soviet Union and other regions worldwide. Case studies discussed within these initiatives demonstrate how mechanochemical approaches can be greener alternatives to conventional chemical methods. Notably, the manuscript also underscores the educational efforts undertaken by these initiatives to further promote mechanochemistry.

 Overall, the Open Letter is concise, well-written, and presents a cohesive overview of selected European activities in mechanochemistry while also providing references to track further initiatives in the field. My assessment of the manuscript is positive, and I support its publication. I only have a few minor points for improvement:

1. The text within the green panel of Figure 3 is difficult to read. Adjusting the hue to a less saturated shade of green or using a bold font may improve readability.
2. The acronym SME, mentioned in the section on IMPACTIVE, is not defined.
3. In the introduction, mechanochemistry is defined as the use of mechanical force to promote chemical transformations. Since none of these mechanical force-induced transformations typically occur at 0 K, it would be beneficial to include one or two sentences that clarify that temperature may also influence mechanochemical processes, albeit to a lesser extent than in conventional methods.
4. When discussing the United Nations’ 17 Sustainable Development Goals, a reference should be provided.

Where applicable, are recommendations and next steps explained clearly for others to follow? (Please consider whether others in the research community would be able to implement guidelines or recommendations and/or constructively engage in the debate)

Yes

Does the article adequately reference differing views and opinions?

Yes

Are all factual statements correct, and are statements and arguments made adequately supported by citations?

Yes

Is the rationale for the Open Letter provided in sufficient detail? (Please consider whether existing challenges in the field are outlined clearly and whether the purpose of the letter is explained)

Yes

Is the Open Letter written in accessible language? (Please consider whether all subject-specific terms, concepts and abbreviations are explained)

Yes

Reviewer Expertise:

Polymer mechanochemistry, organic chemistry, supramolecular chemistry, polymer materials, stimuli-responsive polymers and materials

I confirm that I have read this submission and believe that I have an appropriate level of expertise to confirm that it is of an acceptable scientific standard.
